# MCAF1 and Rta-Activated BZLF1 Transcription in Epstein-Barr Virus

**DOI:** 10.1371/journal.pone.0090698

**Published:** 2014-03-05

**Authors:** Ting-Yu Lin, Ya-Yun Chu, Ya-Chun Yang, Shih-Wei Hsu, Shih-Tung Liu, Li-Kwan Chang

**Affiliations:** 1 Department of Biochemical Science and Technology, College of Life Science, National Taiwan University, Taipei, Taiwan; 2 Molecular Genetics Laboratory, Department of Microbiology and Immunology, Chang-Gung University, Taoyuan, Taiwan; Ecole Normale Supérieure de Lyon, France

## Abstract

Epstein-Barr virus (EBV) expresses two transcription factors, Rta and Zta, which are involved in the transcriptional activation of EBV lytic genes. This study sought to elucidate the mechanism by which Rta activates transcription of the Zta-encoding gene, BZLF1, through the ZII element in the gene promoter. In a DNA affinity precipitation assay, ATF2 was found to associate with an Rta-interacting protein, MCAF1, at the ZII element. The interaction between Rta, MCAF1, and ATF2 at the same site in the ZII region was further verified in vivo by chromatin immunoprecipitation assay. The complex appears to be crucial for the activation of BZLF1 transcription, as the overexpression of two ATF2-dominant negative mutants, or the introduction of MCAF1 siRNA into 293T cells, were both found to substantially reduce Rta-mediated transcription levels of BZLF1. Moreover, this study also found that the Rta-MCAF1-ATF2 complex binds to a typical AP-1 binding sequence on the promoter of BMRF2, a key viral gene for EBV infection. Mutation of this sequence decreased Rta-mediated promoter activity significantly. Taken together, these results indicate a critical role for MCAF1 in AP-1-dependent Rta activation of BZLF1 transcription.

## Introduction

Epstein-Barr virus (EBV) is a human herpesvirus that infects lymphoid and epithelial cells. During the switch from latency to the lytic cycle, EBV expresses two transcription factors, Rta and Zta, which activate the transcription of EBV lytic genes [1]–[4]. These two factors are crucial to lytic progression, as infectious EBV particles cannot be produced in the absence of either factor [5]. It is known that Rta binds to the Rta-response element (RRE) to activate viral promoters [2], [6], [7]. Rta also activates Sp1-dependent transcription via its interaction with an Sp1-binding protein, MCAF1 [8]. Zta, a member of the b-ZIP family, is encoded by the viral gene BZLF1, and regulates EBV lytic replication via binding to *oriLyt* and recruitment of viral replication factors [9], [10]. Emerging evidence suggests that Zta binds to canonical Zta-response elements (ZRE) in viral promoters, with a preference for CpG-methylated ZREs (meZRE), such as those found in the promoters of BBLF4, BMRF1 and BALF5 [11]. During the switch to lytic phase, Zta induces chromatin remodeling at target promoters, and can bind to meZRE in viral promoters without requiring active DNA demethylation [12], suggesting that EBV relies on Zta to reverse epigenetic silencing for induction of the lytic cycle [13]. Additionally, Zta acts in concert with Rta via MCAF1 to activate lytic promoters that contain ZREs [14].

Since Zta is crucial to the switch from latency to lytic progression in EBV, the regulation of the BZLF1 promoter (Zp) has been examined extensively [15]. The *cis*-acting elements of Zp, which are sufficient for basal and induced transcriptional activities, are located within the region of nucleotides (nt) −221 to +12, relative to the transcriptional start site [16], [17]. Previous research has identified three positive *cis*-acting regulatory elements in Zp, respectively known as ZI, ZII, and ZIII [15]. Zta autoregulates its promoter via binding to ZIIIA and ZIIIB [16]. The ZII element is a cyclic AMP response element-like motif that binds CREB, XBP-1, ATF family members, C/EBPs, and AP-1 family proteins [18]–[21]. Previous research has indicated that Rta activates the transcription of BZLF1 through the ZII region, despite the fact that this region does not contain an RRE [22]. Rta was also found to activate p38 signaling, which is known to induce ATF2 phosphorylation and BZLF1 transcription through an undetermined mechanism [22]. Alternatively, Lee et al. have shown that Rta interacts with BRAP2 to prevent the binding of KSR1, resulting in the phosphorylation of ATF2 and the activation of BZLF1 transcription [23]. The results of these studies suggest that the activation of Zp by Rta is associated with ATF2 phosphorylation. This study found that Rta, MCAF1 and ATF2 form a complex at the ATF2-binding sequence in the ZII element to activate BZLF1 transcription, providing the first evidence of MCAF1 involvement in Rta-mediated transcriptional activation of BZLF1.

## Materials and Methods

### Cell lines and EBV lytic induction

P3HR1 (ATCC HTB-62), a Burkitt's lymphoma cell line containing EBV [24], was cultured in RPMI 1640 medium containing 10% fetal calf serum. 293T cells (ATCC CRL-11268) were cultured in Dulbecco's modified Eagle's medium (DMEM) containing 10% fetal calf serum. P3HR1 cells were treated with 30 ng/ml 12-*O*-tetradecanoylphorbol-13-acetate (TPA) and 3 mM sodium butyrate to induce the lytic cycle, according to a method described previously [25], [26].

### Plasmids

Plasmids pcDNA-MCAF1, pEGFP-MCAF1-N, pCMV-R, and pNS3 were described previously [8], [23], [27], [28]. Plasmids pGEX-ATF2, pET-ATF2 and pEGFP-ATF2, all expressing full-length ATF2, were constructed by inserting a PCR fragment that was amplified with primers

5′-CGCGGATCCATGAAATTCAAGTTACA and

5′-CCGCTCGAGTCAACTTCCTGAGGGCTG, using pRSV-ATF2 [29] as a template to respectively clone ATF2 into the *Bam*HI-*Sal*I sites in pGEX-4T1 (Amersham Biosciences), the *Bam*HI-*Xho*I sites in pET32a (Novagen), and the *Bgl*II-*Sal*I sites in pEGFP-C1 (Clontech). Plasmids pET-ATF2-N1 and pET-ATF2-N2, which respectively contain His-tagged ATF2 regions from amino acids 1 to 119 or 120 to 323, were constructed by inserting PCR-amplified fragments into the *Bam*HI-*Xho*I sites in pET-32a. Plasmids pET-ATF2-C and pEGFP-ATF2-C,which respectively express the His-tagged and GFP-tagged ATF2 C-terminal region from amino acids 324 to 505, were constructed by inserting a PCR-amplified fragment into the *Bam*HI-*Xho*I sites in pET32a or *Bgl*II-*Sal*I sites in pEGFP-C3. In addition, plasmid pEGFP-ATF2 (T69A/71A), which encodes a mutant ATF2 in which two phosphorylation sites, Thr-69 and Thr-71, were replaced by alanine, was also constructed. Plasmids pEGFP-MCAF1-DM1, pEGFP-MCAF1-M, and pEGFP-MCAF1-DM2, which respectively encode regions in MCAF1 from amino acids 562 to 817, 818 to 1153, and 1154 to 1270, were constructed by inserting PCR-amplified fragments into pEGFP-C1 at the *Hin*dIII-*Sal*I sites. Plasmid pET-MCAF1, used for expressing His-MCAF1 in *E. coli*, was kindly provided by Dr. H. Saitoh [30]. The region between nt −70 and +113 in Zp was amplified by PCR and inserted into the *Xh*oI and *Hin*dIII sites in pGL2-Basic (Promega) to generate pNS1-ZII. Plasmid pNS1-ZIIM contains the same PCR-amplified sequence as pNS1-ZII, but with the ATF2 binding site modified from 5′-TG**ACAT**CA to 5′-TG**GATC**CA. To generate plasmid pBMRF2-AP1, the region between nt -125 and +37 in the BMRF2 promoter was amplified by PCR, using primers 5′-CCGCTCGAGTGACGTCAGCGGGGGAC and 5′-CCCAAGCTTGACTGACCGTATGTCTGG, and then inserted into the *Xho*I and *Hin*dIII sites in pGL2-Basic. Plasmid pBMRF2-mAP1 contains the same sequence as pBMRF2-AP1, but with the ATF2 binding site changed from 5′-**TG**ACGTCA to 5′-**CA**ACGTCA.

### DNA-affinity precipitation assay (DAPA)

DAPA was performed according to a method described earlier [14]. A biotin-labeled probe containing an ATF2-binding sequence, ZII (5′-CCGCTCGAGCATGACATCACAGAG), was utilized to demonstrate the binding of Rta, MCAF1, and ATF2 to the ATF2-binding site in the ZII element. The 5′-ACAT sequence in the ATF2 site was modified to 5′-GATC for a mutant probe, mZII. A cell lysate prepared from P3HR1 cells treated with sodium butyrate and TPA was mixed with 0.2 µg of biotinylated probe, in a binding buffer containing 60 mM KCl, 12 mM HEPES (pH 7.9), 4 mM Tris-HCl, 5% glycerol, 0.5 mM EDTA, 1 mM dithiothreitol, and 10 µg/ml each of leupeptin, aprotinin, and 4-(2-aminoethyl)-benzenesulfonyl fluoride. After incubating on ice for 45 min, the DNA-protein complex was incubated with 30 µl of Streptavidin MagneSphere Paramagnetic particles (Promega), which were pre-equilibrated in the binding buffer for 1 h at 4°C. The DNA-protein complex was then washed three times with the binding buffer, after which 2X electrophoresis sample buffer was added to the precipitated DNA-protein complex and the solution boiled for 5 min to dissociate the proteins. Finally, the proteins were separated by SDS-polyacrylamide gel electrophoresis, and detected using immunoblotting with specific antibodies.

### Immunoprecipitation

293T cells (1×10^7^) were transfected with plasmids using Turbofect (Thermo). Cell lysates were prepared 24 h after transfection using HEPES buffer (12 mM HEPES and 0.5% Nonidet P-40). Proteins in the lysate were immunoprecipitated with anti-Rta (Argene), anti-MCAF1 (Invitrogen), anti-ATF2 (Abcam), or anti-Flag (Sigma) antibodies. Protein-A/G agarose beads (Oncogene) were then added to the lysate. After shaking for 1 hr at 4°C, the beads were collected by centrifugation and washed three times. Proteins bound to the beads were eluted by adding 2X electrophoresis sample buffer, and analyzed by immunoblotting.

A cell lysate was also prepared from P3HR1 cells that had been treated with TPA and sodium butyrate for 24 h, to examine the interaction between Rta, MCAF1 and ATF2. Immunoprecipitated proteins were detected by immunoblotting using appropriate antibodies.

### GST pull-down assay

GST and GST-ATF2 were purified from *E. coli* BL21(DE3) using glutathione-Sepharose 4B beads (Amersham Biosciences), according to a method described previously [27].

### Immunoblot analysis

Proteins in SDS-polyacrylamide gels were electrotransferred to Hybond C membrane at 90 V for 1 h and probed using appropriate antibodies. Supersignal West Pico chemiluminescent substrate (Pierce) was used to visualize the proteins on the membrane. Antibodies used included anti-Rta, anti-ATF2, anti-MCAF1, anti-His (Sigma), anti-GST (Santa Cruz), anti-GFP (Santa Cruz), anti-Flag, and anti-α-tubulin (Sigma).

### Chromatin immunoprecipitation (ChIP) assay

The ChIP assay was performed according to a method described previously [31]. P3HR1 cells (1×10^7^) were treated with TPA and sodium butyrate to induce the expression of Rta and Zta. Formaldehyde-fixed DNA-protein complexes were immunoprecipitated with anti-IgG, anti-Rta, anti-ATF2, or anti-MCAF1 antibodies. The presence of specific DNA fragments in the precipitates was detected by qPCR, as described elsewhere [14]. Primers used for amplification included 5′-CCGCTCGAGCATGACATCACAGAG and 5′-CAAAAGCTTGTACAAAAGGC for the BZLF1 promoter; 5′-GCCGGAAAGACGCTAAAGAA and 5′- TGGCGGCCATCACTACTGA for the BcLF1 intergenic region; and 5′-GCGTGCCAATCTTGAGGTTT and 5′-ACTAAGATCCAACGGCAGGTC for the BMRF2 promoter. Standard curves were generated using serial dilutions of input DNA (1000, 100, 10, 1 and 0.1%). The Ct value of each reaction was quantified against the standard curve.

### Transient transfection and luciferase assay

Plasmid DNA or MCAF1 siRNA (Invitrogen; primer number: 130189C06) was transfected into 293T cells using Turbofect. To examine promoter activities, cells were harvested and lysed in lysis buffer, and luciferase assays were performed according to a method described earlier [32]. Each transfection experiment was performed three times, and all samples in each experiment were prepared in duplicate.

## Results

### Rta activation of BZLF1 transcription occurs via the ATF2-binding site in Zp

**Figure 1:**
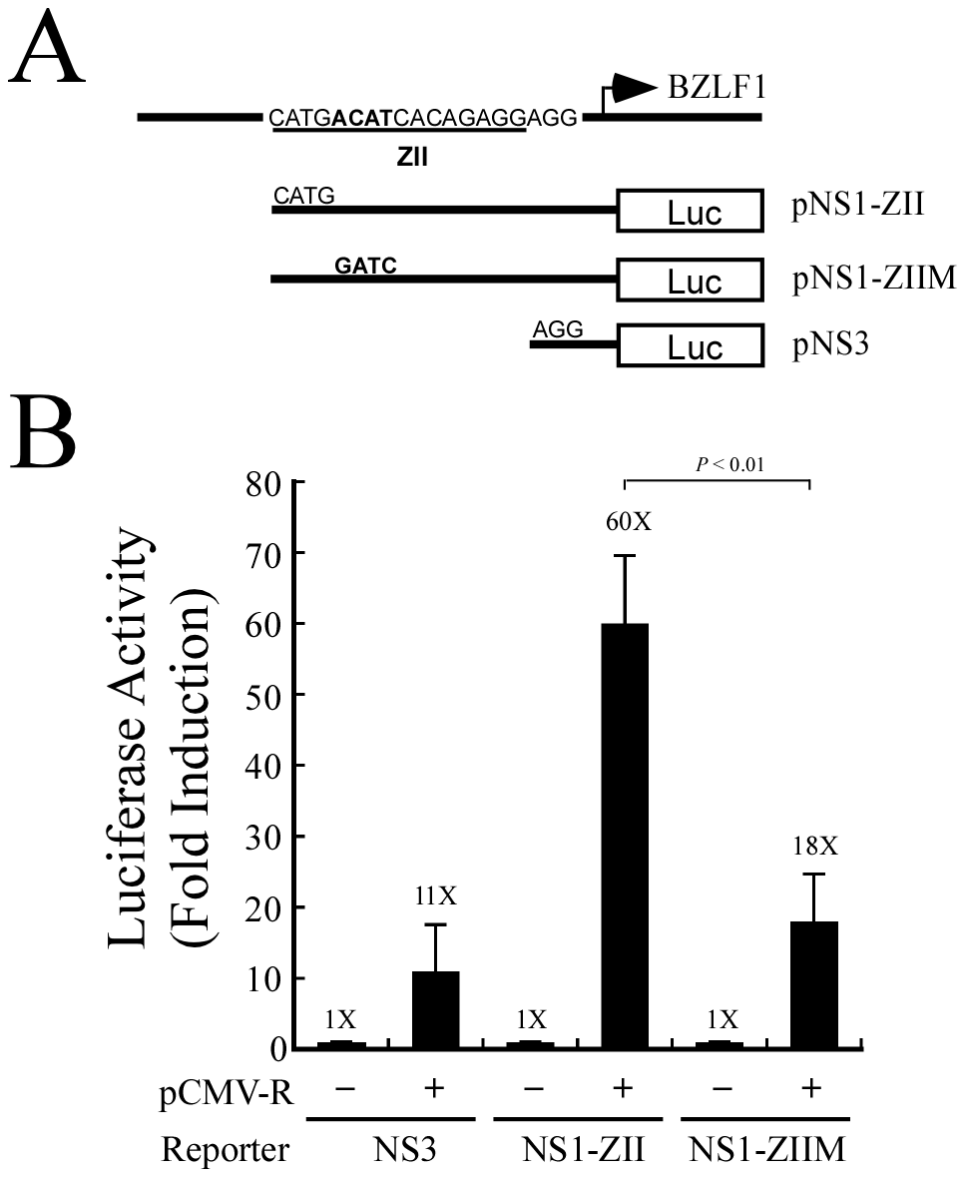
Transcriptional activation of Zp by Rta. (A) Three reporter plasmids, pNS1-ZII, pNS1-ZIIM, and pNS3, were used in this transient transfection experiment. (B) 293T cells were cotransfected with pCMV-R, and the respective reporter plasmids. Luciferase activities were determined at 24 h after transfection. Each transfection experiment was performed three times, and all samples were prepared in duplicate. The *p* value from each experiment was derived using Student's t-test. Luc: luciferase gene.

Rta is known to activate the transcription of BZLF1 via the ZII region in Zp, although such activation is not mediated through a direct binding of Rta to the ZII element [22]. To elucidate how Rta activates Zp activity through ZII, this study generated three reporter plasmids, pNS1-ZII, pNS1-ZIIM, and pNS3. Plasmid pNS1-ZII contained the region from nt −70 to +113 in Zp, including ZII and the TATA box. Plasmid pNS1-ZIIM contained the same region, but with a modified ATF2-binding site (Fig. 1A). Plasmid pNS3 contained the sequence from nt −54 to +113 in Zp, but excluded ZII (Fig. 1A). 293T cells were cotransfected with pCMV-R, which expresses Rta, and the respective reporter plasmids. Luciferase activities were then examined at 24 h after transfection. Results revealed that Rta increased pNS3 promoter activity 11-fold, which can be attributed to basal transcriptional activation by Rta and TAF4 (Fig. 1B) [33]. Interestingly, Rta increased pNS1-ZII promoter activity 60-fold (Fig. 1B); but in pNS1-ZIIM (with a mutated ATF2 site), promoter activity was only increased 18-fold, comparable to basal levels (Fig. 1B). These results show that the ATF2-binding sequence in ZII is critical for transcriptional activation by Rta.

### Coimmunoprecipitation of ATF2, MCAF1 and Rta

**Figure 2:**
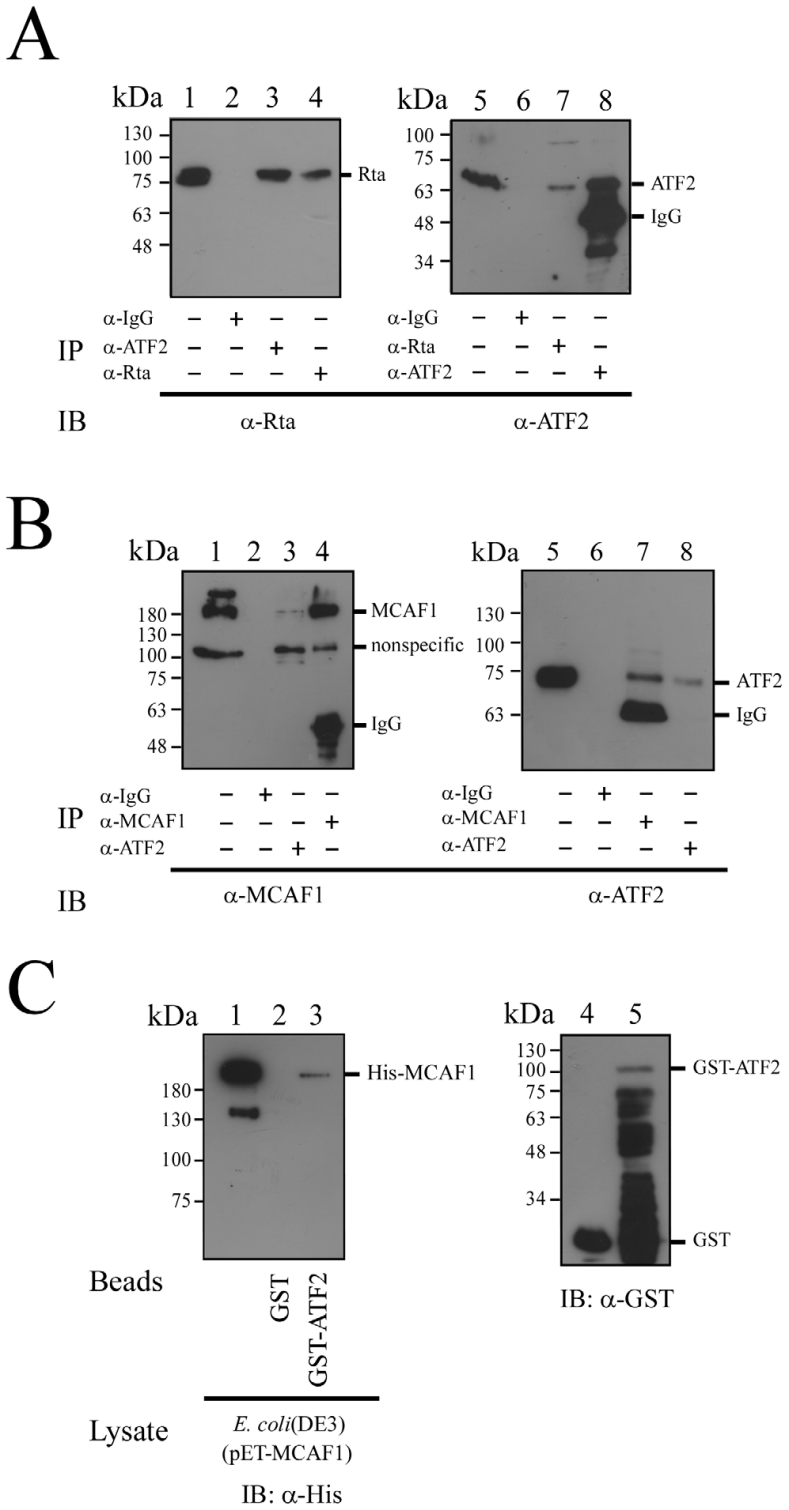
Interaction between Rta, MCAF1 and ATF2. P3HR1 cells were treated with TPA and sodium butyrate for 24(A) Proteins in the lysate were immunoprecipitated with anti-ATF2 antibody (lanes 3, 8), anti-Rta antibody (lanes 4, 7) or anti-IgG antibody (lanes 2, 6). Immunoblot analysis was performed using anti-Rta (lanes 1–4) and anti-ATF2 antibodies (lanes 5–8). (B) Proteins in the lysate from P3HR1 cells were immunoprecipitated with anti-IgG antibody (lanes 2, 6), anti-ATF2 antibody (lanes 3, 8), or anti-MCAF1 antibody (lanes 4, 7). Immunoprecipitated proteins were detected by immunoblotting, using anti-MCAF1 antibody (lanes 1–4) or anti-ATF2 antibody (lanes 5–8). Lanes 1 and 5 in (A) and (B) were loaded with 1% of total protein from the cell lysate. (C) GST-ATF2 (lane 5) or GST (lane 4) was added to the lysate prepared from *E. coli* BL21(DE3)(pET-MCAF1) (lane 1). Proteins bound to GST-ATF2 were pulled down by glutathione-Sepharose beads, and analyzed by immunoblotting (IB) with anti-His antibody (lanes 1–3). Lane 1 was loaded with 1% of cell lysate. GST or GST-ATF2 bound to glutathione-Sepharose beads were analyzed by immunoblotting (IB) with anti-GST antibody (lanes 4, 5).

Since Rta interacts with Zta via MCAF1 [14], this study investigated whether similar interactions with ATF2 at the ZII region were involved in the activation of Zp. A coimmunoprecipitation study was performed, using P3HR1 cells that had been treated with TPA and sodium butyrate for 24 h. The results showed that Rta in cell lysates (Fig. 2A, lane 1) could be immunoprecipitated by anti-Rta antibody (Fig. 2A, lane 4), and coimmunoprecipitated by anti-ATF2 antibody (Fig. 2A, lane 3). A parallel experiment found that ATF2 in cell lysates (Fig. 2A, lane 5) could be immunoprecipitated by anti-ATF2 antibody (Fig. 2A, lane 8), and coimmunoprecipitated by anti-Rta antibody (Fig. 2A, lane 7). However, Rta and ATF2 were not immunoprecipitated by anti-IgG antibody (Fig. 2A, lanes 2, 6). These results confirm the interaction between Rta and ATF2 during lytic activation. A similar experiment revealed that MCAF1 in cell lysates (Fig. 2B, lane 1) could be immunoprecipitated by anti-MCAF1 antibody (Fig. 2B, lane 4), and coimmunoprecipitated by anti-ATF2 antibody (Fig. 2B, lane 3). ATF2 in cell lysates (Fig. 2B, lane 5) was also immunoprecipitated by anti-ATF2 antibody (Fig. 2B, lane 8), and coimmunoprecipitated by anti-MCAF1 antibody (Fig. 2B, lane 7). However, anti-IgG antibody did not immunoprecipitate either MCAF1 or ATF2 (Fig. 2B, lanes 2, 6). Together, these results demonstrate that ATF2 form a complex with MCAF1 and Rta during EBV lytic activation.

Additionally, this study performed a GST-pulldown assay, by adding the *E. coli* BL21(DE3)(pET-MCAF1) lysate to GST- or GST-ATF2-glutathione Sepharose beads. Results showed that bacterially expressed His-MCAF1 was retained by GST-ATF2-glutathione-Sepharose, but not by GST- glutathione-Sepharose beads (Fig. 2C, lanes 2, 3), indicative of a direct interaction between MCAF1 and ATF2.

### Binding of the ATF2-MCAF1-Rta complex to an ATF2 site

**Figure 3:**
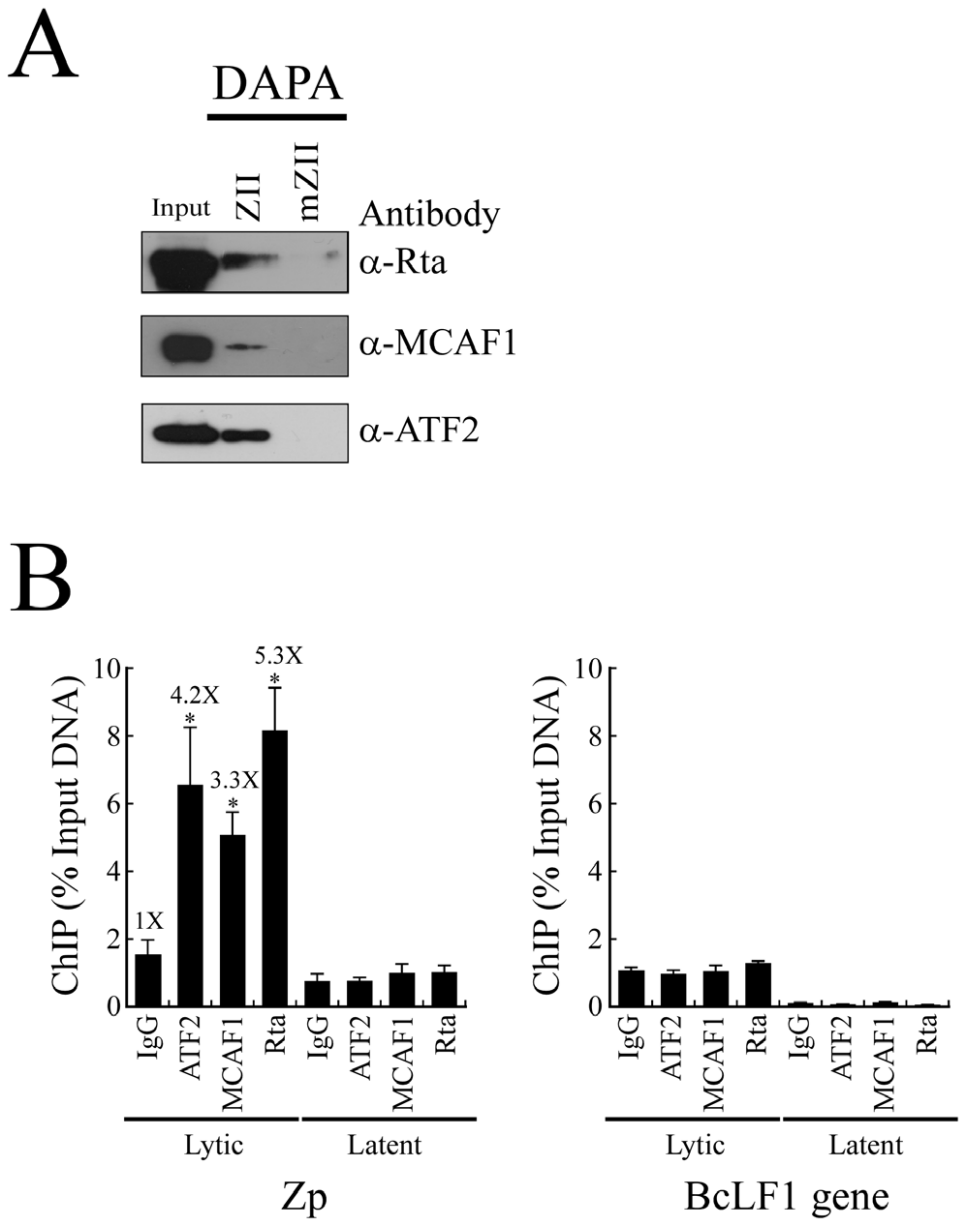
Binding of the Rta-MCAF1-ATF2 complex to the ATF2-binding site in Zp. (A) A biotin-labeled double-stranded ZII probe, containing the ATF2-binding sequences in Zp, was added to a lysate prepared from P3HR1 cells treated with TPA and sodium butyrate. A mutant probe, mZII, which contains a mutated ATF2-binding sequence, was used as a negative control. Proteins bound to the probes were captured using streptavidin magnetic beads, and were then extracted and detected by immunoblotting with anti-Rta, anti-MCAF1, and anti-ATF2 antibodies. Input lanes were loaded with 5% of the cell lysate. DAPA: DNA-affinity precipitation assay. (B) P3HR1 cells treated with TPA and sodium butyrate for 48 h (lytic), or DMSO (latent), were analyzed by ChIP assay using anti-Rta, anti-MCAF1 and anti-ATF2 antibodies, with anti-IgG antibody used as a negative control. The binding capabilities of ATF2, MCAF1, and Rta to the ZII region in Zp were examined by qPCR. qPCR with primers specific for the BcLF1 intergenic region was used as a negative control. The error bar represents standard error. The *p* values from each experiment were derived using the Student's t- test. * *p<*0.05.

To examine whether Rta forms a complex with MCAF1 and ATF2, a DNA affinity precipitation assay (DAPA) was performed. A double-stranded 24-bp biotin-labeled probe containing the ATF2-binding sequence of the ZII region in Zp, and another probe containing a mutated ATF2 sequence (mZII), were added separately to lysates prepared from TPA and sodium butyrate-treated P3HR1 cells. Immunoblot analysis revealed that Rta, MCAF1 and ATF2 bound to the ZII probe but not the mZII probe, indicating that the three proteins may interact to form a complex that binds to the ATF2-binding sequence in the ZII element (Fig. 3A). A ChIP assay was then performed to confirm the binding of ATF2, MCAF1 and Rta to Zp, using P3HR1 cells that had been treated with TPA and sodium butyrate for 48 h to induce the EBV lytic cycle. Cells treated with DMSO were used as a control. After crosslinking, the DNA-protein complex was immunoprecipitated using anti-Rta, anti-MCAF1, and anti-ATF2 antibodies. qPCR, with primers specific to the Zp sequence, was subsequently performed. The amounts of Zp captured by anti-ATF2, anti-MCAF1 and anti-Rta antibodies were respectively 4.2, 3.3, and 5.3 times more than the amounts that were immunoprecipitated by anti-IgG antibody (Fig. 3B, lytic). Moreover, only a background level of Zp DNA was immunoprecipitated by anti-ATF2, anti-MCAF1, anti-Rta, or anti-IgG antibodies, when P3HR1 cells were not treated with sodium butyrate and TPA (Fig. 3B, latent). Additionally, primers that were specific for the BcLF1 intergenic region were used as a negative control, and results showed that only a background level of BcLF1 DNA was immunoprecipitated by anti-ATF2, anti-MCAF1, anti-Rta, or anti-IgG antibodies, regardless of whether P3HR1 cells were previously treated with sodium butyrate and TPA or not (Fig. 3B). Taken together, these experiments provide *in vivo* confirmation regarding the binding of ATF2, MCAF1, and Rta to the ZII region in Zp after lytic induction.

### Mapping the interaction domains in MCAF1 and ATF2

**Figure 4:**
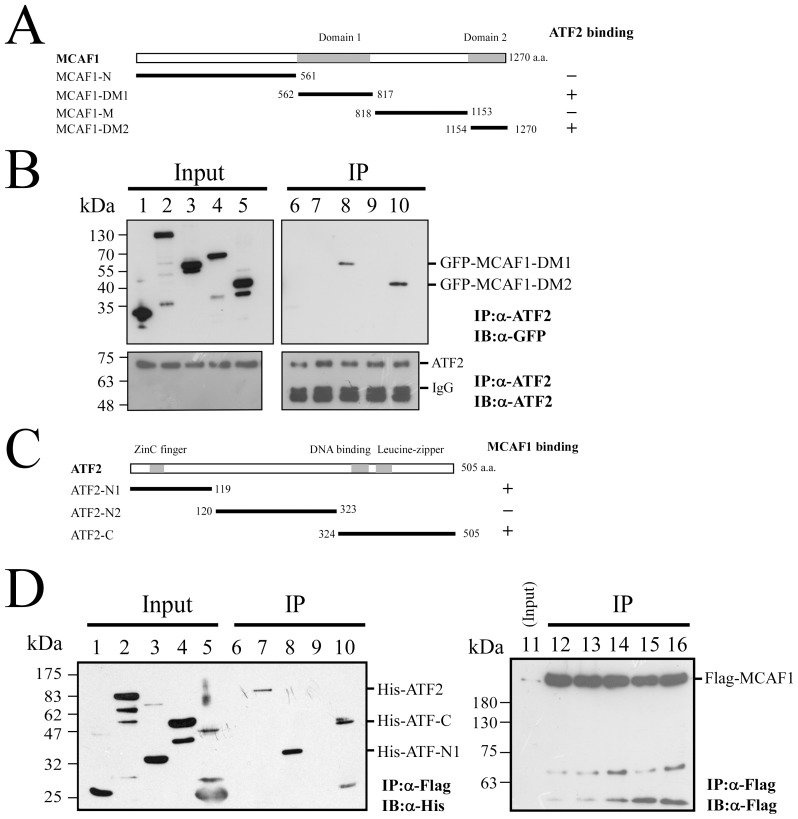
Mapping the interaction domains in MCAF1 and ATF2. Plasmids expressing GFP-MCAF1-N (lanes 2, 7), GFP-MCAF1-DM1 (lanes 3, 8), GFP-MCAF1-M (lanes 4, 9), or GFP-MCAF1-DM2 (lanes 5, 10) were transfected into 293T cells. Plasmid pEGFP-C1 (lanes 1, 6) transfectants were used as a control (A). After transfection, cells were treated with TPA, and proteins in the lysates were immunoprecipitated (IP) using anti-ATF2 antibody. Detection by immunoblotting (IB), using anti-GFP or anti-ATF2 antibodies, was subsequently performed (B). Additionally, lysates prepared from E. coli expressing either His-ATF2 (lanes 2, 7), His-ATF2-N1 (lanes 3, 8), His-ATF2-N2 (lanes 4, 9), or His-ATF2-C (lanes 5, 10) (C) were mixed with the lysates prepared from 293T cells transfected with pcDNA-MCAF1 (lane 11). Proteins immunoprecipitated with anti-Flag antibody (lanes 12–16) were detected by immunoblotting with anti-His (lanes 6–10) or anti-Flag (lanes 12–16) antibodies. E. coli BL21 (pET32a) lysate was used as a negative control (lanes 1, 6) (D). Input lanes in 4B and 4D were loaded with 1% of the cell lysate.

Different segments of MCAF1 were fused with GFP, in order to determine ATF2-interacting regions. Plasmids encoding GFP-MCAF1-N, GFP-MCAF1-DM1, GFP-MCAF1-M, or GFP-MCAF1-DM2 (Fig. 4A) were transfected into 293T cells. An empty vector, pEGFP-C1, which expresses GFP, was also transfected, and used as a negative control. MCAF1 domains 1 and 2 are the regions known to interact with transcription factors [28]. Immunoblot analysis revealed that ATF2 in cell lysates (Fig. 4B, lanes 1–5) was immunoprecipitated by anti-ATF2 antibody (Fig. 4B, lanes 6–10). Moreover, GFP-MCAF1-DM1 and GFP-MCAF1-DM2 were present in cell lysates and could be coimmunoprecipitated by anti-ATF2 antibody (Fig. 4B, lanes 8, 10). GFP, GFP-MCAF1-N, and GFP-MCAF1-M, although detectable in cell lysates (Fig. 4B, lanes 1, 2, and 4), were not coimmunoprecipitated by anti-ATF2 antibody (Fig. 4B, lanes 6, 7 and 9), indicating that domain 1 and domain 2 in MCAF1 interact with ATF2. An additional experiment, using ATF2 truncated mutants, was performed to define the region in ATF2 that binds to MCAF1. The His-tagged ATF2 truncated proteins were expressed in E. coli and were detected in cell lysates by immunoblotting with anti-His antibody (Fig. 4D). His-tagged ATF2 truncated proteins were then added to lysates from 293T cells that had been transfected with pcDNA-MCAF1, which encodes Flag-MCAF1. Immunoblot analysis showed that Flag-MCAF1 in cell lysates (Fig. 4D, lane 11) was immunoprecipitated by anti-Flag antibody (Fig. 4D, lanes 12–16). Proteins bound to MCAF1 were also immunoprecipitated, and detected by immunoblotting using anti-His antibody. Results showed that His-ATF2, His-ATF2-N1, and His-ATF2-C (Fig. 4D, lanes 7, 8, and 10), were able to bind with MCAF1, but not His-ATF2-N2 (Fig. 4D, lane 9).

### Involvement of MCAF1 in the interaction between ATF2 and Rta

**Figure 5:**
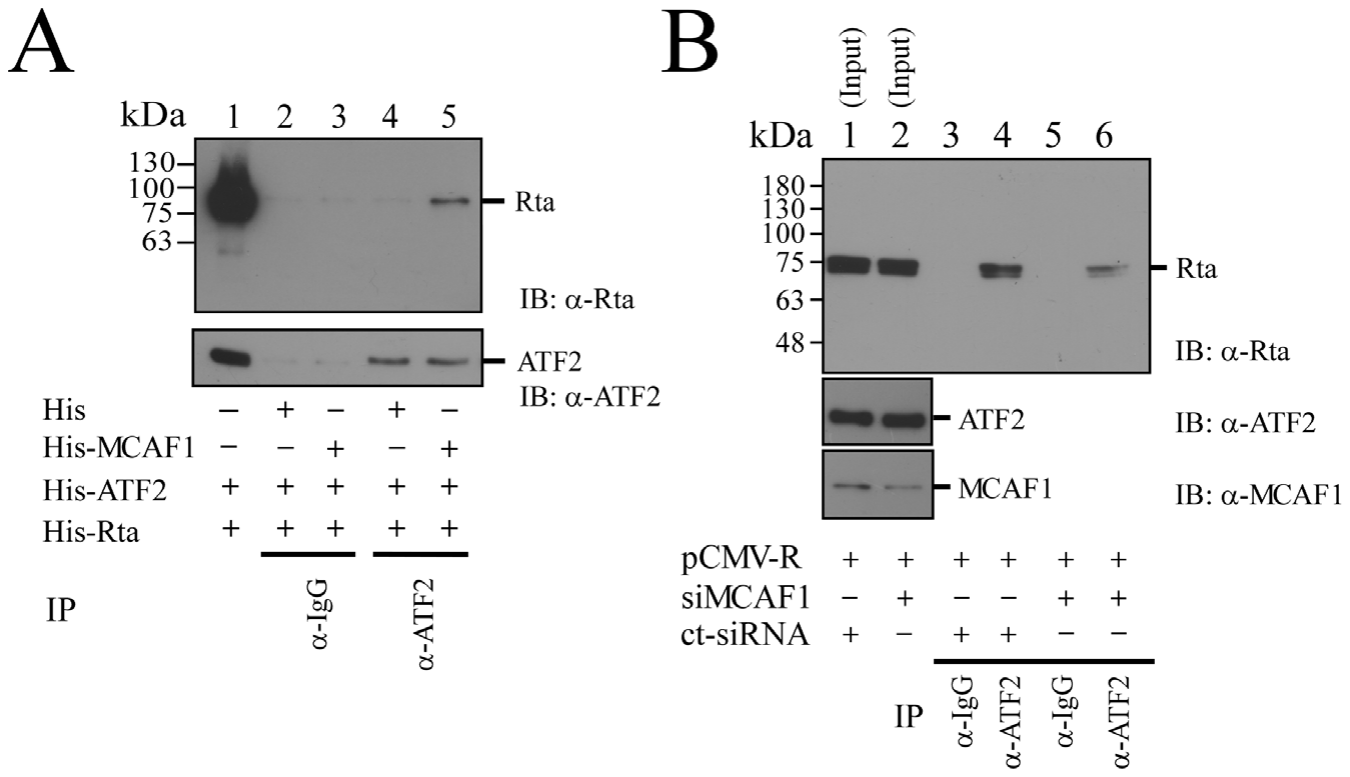
Involvement of MCAF1 in the interaction between ATF2 and Rta. (A) His-tagged proteins, including Rta, ATF2, and MCAF1, were expressed in E. coli BL21(DE3). Lysates were mixed in different combinations for immunoprecipitation (IP) using anti-ATF2 antibody, and proteins bound to protein A/G agarose beads were then detected by immunoblotting (IB) using anti-Rta or anti-ATF2 antibodies. Lane 1 was loaded with 1% His-Rta. The reaction was also conducted using anti-IgG as a control. (B) 293T cells were cotransfected with pCMV-R and either MCAF1 siRNA or control siRNA. After transfection, cells were immunoprecipitated (IP) by anti-ATF2 (lanes 4, 6) or anti-IgG antibody (lanes 3, 5), and detected by immunoblotting (IB) using anti-Rta antibody. The effect of MCAF1 siRNA on the expression of MCAF1 was examined by immunoblotting using anti-MCAF1 and anti-ATF2 antibodies (lanes 1, 2). Lanes 1 and 2 were loaded with 1% of total protein from cell lysates.

In order to examine the involvement of MCAF1 in ATF2-Rta interactions, varying combinations of bacterial lysates containing His-ATF2, His-MCAF1, or His-Rta were used in immunoprecipitation. Adding anti-ATF2 antibody to the mixtures did not immunoprecipitate His-Rta when the reaction did not include His-MCAF1 (Fig. 5A, lane 4), demonstrating that ATF2 does not bind to Rta directly. However, Rta was coimmunoprecipitated by anti-ATF2 antibody if His-MCAF1 was present (Fig. 5A, lane 5). Anti-IgG was used as a control, and did not immunoprecipitate Rta or ATF2, regardless of whether His-MCAF1 was present or not (Fig. 5A, lanes 2, 3). A further coimmunoprecipitation assay was performed in 293T cells, in order to confirm the necessity of MCAF1 for complex formation between Rta and ATF2. 293T cells were cotransfected with pCMV-R and either MCAF1 siRNA or control siRNA. Results showed that Rta in cell lysates (Fig. 5B, lanes 1, 2) could be immunoprecipitated by anti-ATF2 antibody (Fig. 5B, lane 4), but silencing the expression of MCAF1 by siRNA (Fig. 5B, lanes 5, 6) reduced the interaction between Rta and ATF2, as compared with results for control siRNA transfectants (Fig. 5B, lanes 4, 6). Anti-IgG antibody was unable to immunoprecipitate Rta or ATF2, regardless of whether MCAF1 was silenced or not (Fig. 5B, lanes 3, 5). These results show that MCAF1 is a key mediator of the interaction between ATF2 and Rta.

### Involvement of MCAF1 and ATF2 in the activation of BZLF1 transcription by Rta

**Figure 6:**
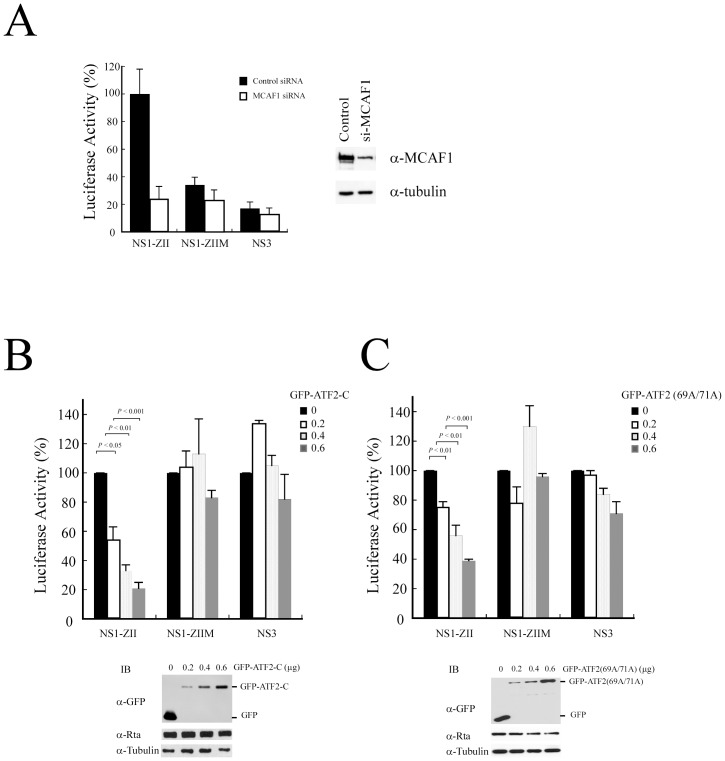
Involvement of MCAF1 in Rta-activated BZLF1 transcription. (A) 293T cells were cotransfected with pCMV-R and a reporter plasmid, pNS1-ZII, pNS1-ZIIM, or pNS3, as well as control siRNA or MCAF1 siRNA. Luciferase activity exhibited by the cells was examined at 48 h after transfection. The reduction of MCAF1 by siRNA was examined by immunoblotting with anti-MCAF1 antibody. Anti-α-tubulin antibody was used as a control. In addition, 293T cells were cotransfected with pCMV-R and a reporter plasmid, pNS1-ZII, pNS1-ZIIM, or pNS3, as well as varying amounts (µg) of pEGFP-ATF2-C (B) or pEGFP-ATF2 (69A/71A) (C). The expression of GFP-ATF2-C (B) or GFP-ATF2 (69A/71A) (C) was examined by immunoblotting, using anti-GFP antibody. Promoter activities were determined at 48 h after transfection. Each transfection experiment was performed at least three times, and all samples were prepared in duplicate. The *p* values from each experiment were derived using the Student's t-test.

To elucidate whether MCAF1 is involved in the transcriptional activation of BZLF1 by Rta, three reporter plasmids, pNS1-ZII, pNS1-ZIIM, and pNS3 were respectively cotransfected with pCMV-R and either MCAF1 siRNA or control siRNA into 293T cells. Silencing the expression of MCAF1 by siRNA (Fig. 6A) reduced Rta-mediated promoter activity in pNS1-ZII by 80% (Fig. 6A). However, MCAF1 siRNA did not appear to affect promoter activity in pNS1-ZIIM and pNS3 (Fig. 6A). The effect of ATF2 on Rta-mediated promoter activity was also examined. The same three plasmids were respectively cotransfected with pCMV-R and varying amounts of pEGFP-ATF2-C (Fig. 6B), which expresses an ATF2 mutant lacking the transactivation domain. Luciferase activity measurements revealed that overexpressing ATF2-C decreased Rta-mediated promoter activity in pNS1-ZII by up to 80%, in a dose-dependent manner. However, expression levels of ATF2-C did not affect promoter activity in pNS1-ZIIM and pNS3 transfectants (Fig. 6B). Similar experiments were performed using pEGFP-ATF2 (69A/71A), which abolished the phosphorylation of ATF2 [34]. As pEGFP-ATF2 (69A/71A) levels increased, Rta-mediated promoter activity for pNS1-ZII was shown to drop in proportion (Fig. 6C), indicating that ATF2 phosphorylation is crucial for Rta-activated BZLF1 transcription.

### Role of MCAF1 and ATF2 in the activation of BMRF2 transcription by Rta

**Figure 7:**
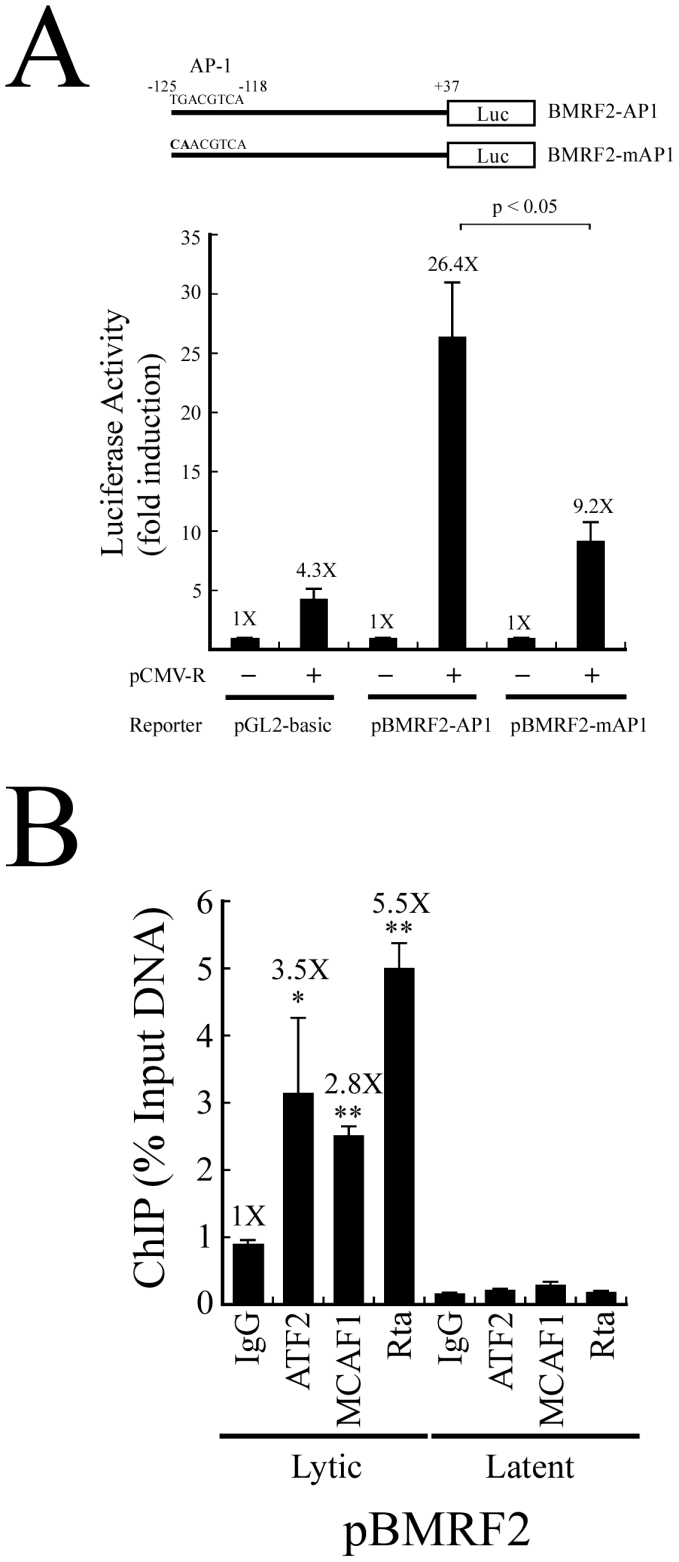
Activation of the BMRF2 promoter by Rta via the AP-1 binding site. (A) The BMRF2 reporter plasmid and a mutant version. 293T cells were cotransfected with pCMV-R and a reporter plasmid, pBMRF2-AP1, pBMRF2-mAP1, or pGL2-Basic. Luciferase activities were examined at 24 h after transfection. (B) P3HR1 cells that had been treated with TPA and sodium butyrate for 48 h (lytic) or DMSO (latent) were analyzed by ChIP assay using anti-Rta, anti-MCAF1 and anti-ATF2 antibodies. Anti-IgG antibody was used as a control. The binding of ATF2, MCAF1 and Rta to the AP-1 sequence in the BMRF2 promoter was examined by qPCR. Error bars represent standard error. The *p* values from each experiment were derived using the Student's t-test. * *p<*0.05, ** *p*<0.001. Luc: luciferase gene.

BMRF2 is an EBV glycoprotein that is crucial for infection of oral epithelial cells [35]. Sequence analysis showed that the BMRF2 promoter contains a typical AP-1 binding sequence. Therefore, this study generated a reporter plasmid, pBMRF2-AP-1, which contains the BMRF2 promoter from nt −125 to +37. This region covers the AP-1 binding sequence and was cloned upstream of the luciferase gene. A mutant plasmid containing a mutated AP-1 site, pBMRF2-mAP1, was used as a control. 293T cells were cotransfected with pCMV-R, pBMRF2-AP-1, or pBMRF2-mAP1, and promoter activities were detected at 24 h after transfection. Results indicated that overexpressing Rta activated the transcription of BMRF2 about 26-fold. However, Rta-activated promoter activity only increased 9-fold when the AP-1-binding site was mutated (Fig. 7A). A ChIP assay was performed to assess the binding of ATF2, MCAF1, and Rta to the BMRF2 promoter. P3HR1 cells were treated with sodium butyrate and TPA for 48 h to induce the expression of EBV lytic proteins. After crosslinking, the DNA-protein complex was immunoprecipitated by anti-IgG, anti-Rta, anti-MCAF1, or anti-ATF2 antibodies. qPCR analysis was then performed, using primers that amplified the AP1-binding sequences in the BMRF2 promoter. The levels of promoter sequences captured by anti-ATF2, anti-MCAF1, and anti-Rta antibodies were 3.5, 2.8, and 5.5 times higher than those immunoprecipitated by anti-IgG antibody. In addition, when cells were treated with DMSO, only a background level of BMRF2 promoter DNA was amplified by qPCR (Fig. 7B). These results demonstrate that ATF2, MCAF1, and Rta were able to bind with the BMRF2 promoter at an AP-1-binding site to activate transcription (Fig. 7B).

## Discussion

Rta activates viral promoters via direct binding to RREs [2], [6], [7]. Rta also interacts with cellular proteins, such as MCAF1, USF, BRAP2, RanBPM, and TAF4, to affect transcription [8], [23], [33], [36], [37]. Earlier studies indicated that Rta activates BZLF1 transcription indirectly, and that such activation is attributable to Rta-induced p38 and ERK signaling, which ultimately lead to the phosphorylation of ATF2 and the transcription of BZLF1 via the ZII element in Zp [22], [23]. The ZII region in Zp is crucial for the lytic activation of EBV by TPA, as TPA is unable to activate Zp promoter activity when the sequence is mutated [17]. This study connects the dots of a novel mechanism in Rta-mediated BZLF1 transcription, by demonstrating that Rta interacts with ATF2 and MCAF1 via the ZII element (Fig. 3 and Fig. 5), resulting in the activation of Zp.

MCAF1, also named AM, was first identified using ATFa as bait in yeast two-hybrid analysis [38]. MCAF1 is known to interact with Sp1, MBD1, and several components of the basal transcription machinery to fine-tune transcription [8], [28]. MCAF1 also interacts with Zta, and is involved in Rta and Zta synergistic activity [14]. Adamson et al. [22] previously showed that Rta does not bind directly to Zp or ATF2, and thus the activation of Zp by Rta must then be mediated via an unknown factor. This study demonstrates that Rta interacts with ATF2 in the presence of His-MCAF1 (Fig. 5A), and knockdown of MCAF1 reduces the interaction of Rta and ATF2 (Fig. 5B). Furthermore, mutation of the ATF2 binding site in the ZII region was also found to abolish the ability of Rta to transactivate Zp (Fig. 1). Together, these results indicate that MCAF1 mediates the interaction between Rta and ATF2.

Earlier studies have shown that transcription factors, including CREB, ATF1, ATF2, c-Jun, and XBP-1, bind to the ZII element [18]–[21]. The fact that MCAF1 interacts with ATF2 (Fig. 2), while both Rta and MCAF1 interact with ATF1, c-jun, c-fos, and Zta (data not shown), suggest that Rta is recruited by AP-1 family proteins via MCAF1 to activate Zp at the ZII region. Furthermore, the activation of Zp by Rta requires phosphorylation of these cellular factors [22], [23]. This study found that the ability of Rta to transactivate Zp is reduced when levels of an ATF2 phosphorylation-deficient mutant increase (Fig. 6), which is consistant with previous findings that ATF2 phosphorylation is required for Rta to activate Zp [22].

Finally, this study demonstrated that Rta activates AP-1-dependent transcription (Fig. 7A). Previous research indicated that Rta increases BMRF2 transcription 2.9-fold, as shown by microarray analysis [39]. Yet it remains unclear as to how Rta activates the BMRF2 promoter. By using sequence analysis, this study identified a canonical AP-1-binding site in the BMRF2 promoter. Rta was able to increase BMRF2 promoter activity 26-fold, but only 9-fold if the AP-1 site was mutated (Fig. 7A), suggesting that AP-1 is involved in Rta-mediated BMRF2 transcription. Rta also forms a complex with ATF2 and MCAF1 on the BMRF2 promoter, as evidenced by a ChIP assay (Fig. 7B). These results indicate that MCAF1 is involved in Rta-mediated BZLF1 and BMRF2 transcription, and suggest that MCAF1 may be associated with the transcriptional activation of other viral promoters that contain AP-1 consensus sequences. Taken together, this highlights the critical role of MCAF1 in EBV lytic development, through its participation in Rta autoregulation [8], Rta and Zta synergistic activities [14], and the activation of Zp. This study also provides evidence of a novel strategy employed by Rta to activate the expression of a broad spectrum of viral genes crucial to EBV biology.
